# In vivo efficacy and safety of artemether–lumefantrine and amodiaquine–artesunate for uncomplicated *Plasmodium falciparum* malaria in Mozambique, 2018

**DOI:** 10.1186/s12936-021-03922-9

**Published:** 2021-10-02

**Authors:** Abel Nhama, Lídia Nhamússua, Eusébio Macete, Quique Bassat, Crizolgo Salvador, Sónia Enosse, Baltazar Candrinho, Eva Carvalho, Arsénio Nhacolo, Arlindo Chidimatembue, Abuchahama Saifodine, Rose Zulliger, Naomi Lucchi, Samaly S. Svigel, Leah F. Moriarty, Eric S. Halsey, Alfredo Mayor, Pedro Aide

**Affiliations:** 1 Centro de Investigação em Saúde de Manhiça (CISM), Maputo, Mozambique; 2 Instituto Nacional de Saúde (INS), Ministério da Saúde, Maputo, Mozambique; 3 Direção Nacional de Saúde Pública, Ministério da Saúde, Maputo, Mozambique; 4 Barcelona Institute for Global Health (ISGlobal), Hospital Clínic - Universitat de Barcelona, Barcelona, Spain; 5 ICREA, Pg. Lluís Companys 23, 08010 Barcelona, Spain; 6 Pediatric Infectious Diseases Unit, Pediatrics Department, Hospital Sant Joan de Déu (University of Barcelona), Barcelona, Spain; 7 Consorcio de Investigación Biomédica en Red de Epidemiología y Salud Pública (CIBERESP), Madrid, Spain; 8 Programa Nacional de Controlo da Malária, Ministério da Saúde, Maputo, Mozambique; 9 World Health Organization, WHO Country Office Maputo, Maputo, Mozambique; 10 United States President’s Malaria Initiative, United States Agency for International Development, Maputo, Mozambique; 11 United States President’s Malaria Initiative, Centers for Disease Control and Prevention, Maputo, Mozambique; 12 Malaria Branch, Centers for Disease Control and Prevention, Atlanta, GA USA; 13 United States President’s Malaria Initiative, Atlanta, GA USA

**Keywords:** Efficacy, Artemether–lumefantrine, Artesunate–amodiaquine, Uncomplicated malaria, Children, Mozambique

## Abstract

**Background:**

Artemisinin-based combination therapy (ACT) has been the recommended first-line treatment for uncomplicated malaria in Mozambique since 2006, with artemether–lumefantrine (AL) and amodiaquine–artesunate (AS–AQ) as the first choice. To assess efficacy of currently used ACT, an in vivo therapeutic efficacy study was conducted.

**Methods:**

The study was conducted in four sentinel sites: Montepuez, Moatize, Mopeia and Massinga. Patients between 6 and 59 months old with uncomplicated *Plasmodium falciparum* malaria (2000–200,000 parasites/µl) were enrolled between February and September of 2018, assigned to either an AL or AS–AQ treatment arm, and monitored for 28 days. A Bayesian algorithm was applied to differentiate recrudescence from new infection using genotyping data of seven neutral microsatellites. Uncorrected and PCR-corrected efficacy results at day 28 were calculated.

**Results:**

Totals of 368 and 273 patients were enrolled in the AL and AS–AQ arms, respectively. Of these, 9.5% (35/368) and 5.1% (14/273) were lost to follow-up in the AL and AS–AQ arms, respectively. There were 48 and 3 recurrent malaria infections (late clinical and late parasitological failures) in the AL and AS–AQ arms, respectively. The day 28 uncorrected efficacy was 85.6% (95% confidence interval (CI) 81.3–89.2%) for AL and 98.8% (95% CI 96.7–99.8%) for AS–AQ, whereas day 28 PCR-corrected efficacy was 97.9% (95% CI 95.6–99.2%) for AL and 99.6% (95% CI 97.9–100%) for AS–AQ. Molecular testing confirmed that 87.4% (42/48) and 33.3% (1/3) of participants with a recurrent malaria infection in the AL and AS–AQ arms were new infections; an expected finding in a high malaria transmission area. Adverse events were documented in less than 2% of participants for both drugs.

**Conclusion:**

Both AL and AS–AQ have therapeutic efficacies well above the 90% WHO recommended threshold and remain well-tolerated in Mozambique. Routine monitoring of therapeutic efficacy should continue to ensure the treatments remain efficacious.

*Trial registration* Clinicaltrials.gov: NCT04370977

**Supplementary Information:**

The online version contains supplementary material available at 10.1186/s12936-021-03922-9.

## Background

Malaria remains one of the largest public health problems in the world. According to the World Health Organization (WHO), approximately 229 million cases of malaria and 409,000 deaths from malaria occurred globally in 2019. The majority of malaria cases (94%) occurred in sub-Saharan Africa, where more than 71 million children aged 1–59 months had malaria in 2019 [1].

In Mozambique, about 9 million malaria cases and about 16,000 malaria deaths (~ 4% of global deaths) were estimated to have occurred in 2019 [1]. According to the 2018 Malaria Indicator Survey (MIS), the prevalence of *Plasmodium falciparum* infection in children under 5 years of age was 39% [2]. One of the key strategies of malaria control in endemic areas is the rapid diagnosis and treatment of malaria with an effective anti-malarial. Resistance of *P. falciparum* to commonly used anti-malarials represents a major obstacle, particularly in Southeast Asia, where evidence for the emergence of resistance to artemisinin derivatives is well-documented [3–7]; recently, emergence of partial resistance to artemisinin was reported in East Africa [8].

In Mozambique, several clinical trials of anti-malarial therapeutic efficacy were conducted and used as a basis for changes in national guidelines for the treatment of malaria [9–13]. The country adopted artemisinin-based combination therapy (ACT) in 2006, and artemether–lumefantrine (AL) and artesunate–amodiaquine (AS–AQ) were introduced as first-line treatment for uncomplicated malaria in 2009. A variety of studies have been conducted since their adoption [9–11, 14], including two in vivo efficacy monitoring efforts, the first one in the period 2011–2012, which showed a day 28 PCR-corrected efficacy of 96.0% for AL and 99.6% for AS–AQ [12]; and the second, in 2015, which documented in four sites a day 28 PCR-corrected efficacy of 98.4% for AL [13].

## Methods

### Study design and sites

**Fig. 1:**
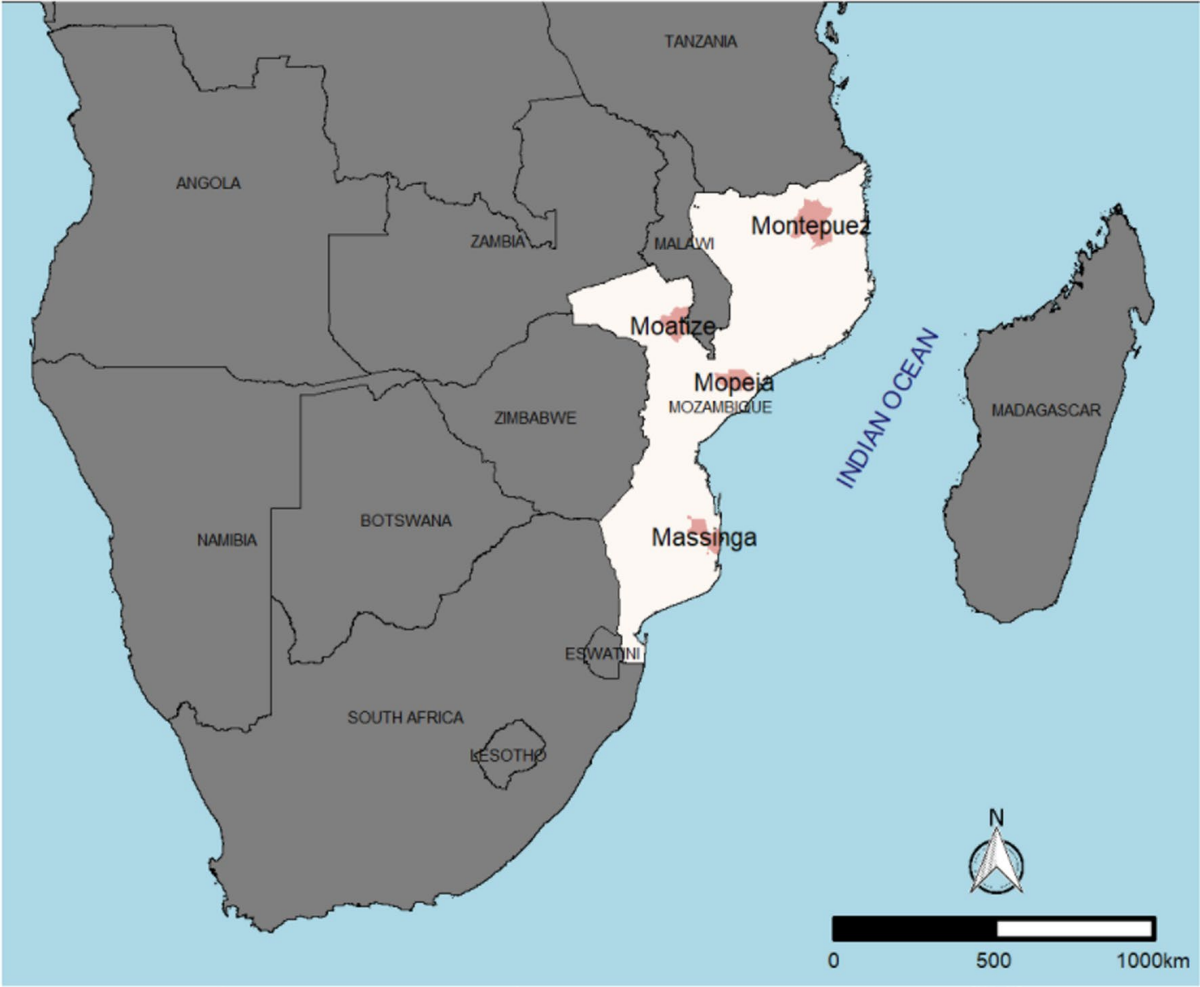
Map of Mozambique with study sites

This study was conducted following the standard WHO in vivo protocol for anti-malarial efficacy surveillance [15]. Two drug combinations, AL and AS–AQ, were tested in three regions (South, Centre, and North) of the country (Fig. 1). The study took place in hospitals or health centres in four sentinel sites across Mozambique: (1) *Hospital Rural de Montepuez*, in Cabo Delgado province (Northern region); (2) *Centro de Saúde de Moatize*, in Tete province (Central region); (3) *Hospital Distrital de Mopeia*, in Zambezia province (Central region); and (4) *Hospital Distrital de Massinga*, in Inhambane province (Southern region). With the exception of Montepuez, these study sites are different from those used in the prior drug efficacy study evaluating both AL and AS–AQ in Mozambique. Parasite prevalence during the 2018 MIS was 57.3% in Cabo Delgado, 29.4% in Tete, 44.3% in Zambezia, and 35.1% in Inhambane [2]. The AL arm began in February 2018, concluded in June 2018, and involved all four sites, while the AS–AQ arm started in June 2018, concluded in September 2018, and was only conducted in three of the four sites (Montepuez, Mopeia, and Massinga) due to financial constraints.

### Study population

Children reporting to the health clinic with febrile illness (≥ 37.5 °C axillary) or a history of fever in the preceding 24 h were screened for eligibility. Children aged 6 to 59 months with microscopically confirmed uncomplicated *P. falciparum* malaria with a body weight ≥ 5 kg, *P. falciparum* mono-infection with an asexual blood density between 2000/µl and 200,000/µl, and the absence of signs of severe malaria as defined by the WHO [16] were eligible for inclusion. Children with mixed malarial infections, haemoglobin < 5 g/dl, severe malnutrition, intake of anti-malarials within the preceding 7 days, ongoing prophylaxis in HIV positive patients with cotrimoxazole or the intake of any other drug with anti-malarial activity, and any serious underlying disease were excluded from this study.

### Study procedures

Enrolled patients were treated with AL or AS–AQ. AL (one tablet containing artemether 20 mg and lumefantrine 120 mg; Coartem™; Novartis, Basel, Switzerland) was administered twice daily for 3 days (six doses in total) with dosage determined according to body weight: one tablet for children 5–15 kg and two tablets for children 15–25 kg. AS–AQ (Coarsucam™; Winthrop; Sanofi Aventis, Paris, France) was administered once daily according to body weight: 25 mg artesunate/67.5 mg amodiaquine for children 4.5–9 kg; 50 mg artesunate/135 mg amodiaquine for children 9–18 kg; 100 mg artesunate/270 mg amodiaquine for children 18–36 kg [17]. Both drugs were administered with food. All treatments were directly supervised for a minimum of 30 min at the health facility. For vomiting occurring within the first 30 min the full treatment dose was readministered. For patients living far away from the health centers, and those for whom direct observation of the evening doses of AL was challenging, admission was offered for the first 3 days of the study.

Antipyretics, such as paracetamol, were used to control fever. In case of development of severe malaria or danger signs, the patient was hospitalized and received parenteral artesunate. Rescue therapy according to national treatment guidelines was also administered in cases of early or late treatment failure [18].

### Sample size calculations

A 5% failure rate was assumed using previous studies from Mozambique indicating that AL and AS–AQ efficacy is above 95% [12, 13]. For a confidence level of 95%, a precision level of ± 5% and loss to follow-up of 20%, a minimum of 88 patients was estimated necessary for recruitment at each of the sites and for each of the study arms (AL: n = 352 at four sites; AS–AQ: n = 264 at three sites).

### Assessment during follow-up

Study participants were followed daily for the first 3 days after initiation of treatment and then weekly until a total of 28 days had passed or at any point in time when the child was sick (unscheduled visit). Patients who were prematurely discontinued from the study (lost to follow-up, withdrawal, or protocol violation) were scheduled for a final visit before or on day 28. If they did not attend the day 28 visit, the outcome of the malaria episode was determined when possible. Vital signs, haemoglobin level, and body temperature were assessed at all visits during the follow-up in the study. Adverse events related to tolerability were recorded and assessed for severity and association with the study medication.

### Laboratory analysis

Thick and thin Giemsa-stained blood slides were prepared before each treatment dose was administered and at every follow-up visit. Slides were examined by two independent certified microscopists. All microscopists participating in the study were certified by the National Health Laboratory Service, receive internal and external quality control evaluations, and have attained microscopy qualifications equivalent to WHO level 1 or 2. If parasites were detected, densities were calculated as the average of the two microscopists’ results. Parasite density, expressed as the number of asexual parasites per µl of blood, was calculated by dividing the number of asexual parasites by the number of white blood cells and then multiplied by an assumed white blood cell density of 6000 per µl. Slides were considered negative if no parasites were seen after examination of 200 oil-immersion fields in a thick blood film. In case of discrepancies in the outcome (i.e., one microscopist detected parasites and the other did not), a third microscopist was engaged. The participant’s result was considered negative if the third reader’s result was negative. *Plasmodium* species determination was made based on assessment of thin films. Haemoglobin was measured by semi-quantitative methods using the “Hemocue” 201 technique. Dried blood spots were collected on filter papers from every patient at enrollment (day 0) and at days 7, 14, 21, and 28 or at any other unscheduled visit. DNA was extracted using QIAamp DNA Mini Kit (Qiagen, Hilden, Germany) at Manhiça Health Research Center Laboratory, Mozambique. Extracted DNA was transported to Malaria Branch Laboratory, Centers for Disease Control and Prevention (CDC), Atlanta, USA. Molecular analysis was conducted by Mozambican investigators under the President’s Malaria Initiative-supported Antimalarial Resistance Monitoring in Africa Network capacity building programme [19]. DNA samples were tested using PET-PCR to confirm the presence of *P. falciparum* [20]. Paired day 0 and day of recurrent malaria samples (for all cases of recurrent parasitemia after day 7) were analysed using seven neutral microsatellite markers (TA1, Poly-α, PfPK2, TA109, TA2490, C2M34 and C3M69) [21]. In addition to the paired samples, an extra 58 samples from the four sites were assessed in order to generate additional background allele frequency data and three laboratory *P. falciparum* strains (3D7, Dd2 and 7G8) were included. Fragment lengths of the microsatellite markers were measured using an ABI 3130 xl Genetic Analyzer (Applied Biosystems, Foster City, CA) and data analysed using GeneMarker software (SoftGenetics, State College, PA). When two or more peaks were observed, minor peaks were reported if they were at least 30% of the major peak.

### Study outcomes

The primary efficacy outcomes were early treatment failure (ETF), late clinical failure (LCF), late parasitological failure (LPF), or adequate clinical and parasitological response (ACPR) at day 28, according to the WHO protocol [15]. Uncorrected day 28 efficacy was calculated by dividing the ACPR by the number with a primary outcome (i.e., ETF, LCF, LPF, and ACPR). PCR-corrected day 28 efficacy was calculated by dividing the ACPR by the sum of ACPR, early treatment failures, and the recrudescent infections; new infections were not included in the numerator or the denominator. Secondary outcomes included 28-day uncorrected ACPR (crude efficacy), safety and tolerability profiles, clearance of parasitemia, gametocytes, and haemoglobin changes from baseline to day 28.

### Data management and statistical analysis

Data were recorded using standardized case report forms as proposed by the WHO [15]. To avoid transcription errors, all questionnaires were double entered into a specific database by two different and independent data entry clerks, using open clinic software (OpenClinica Enterprise—Electronic Data Capture Software for Clinical Trials version 3.2, OpenClinica LLC, Waltham, MA, USA). A previously validated Bayesian (i.e., probabilistic) algorithm accounting for the background prevalence of alleles was used to assign each late treatment failure a posterior probability of recrudescence [22]. Samples with a posterior probability of recrudescence of 50% or above were defined as recrudescences, those with a probability less than 50% were defined as new infections and were used to calculate per-protocol and cumulative efficacy. Undetermined late treatment failures were assigned the average posterior probability of recrudescence [22]. Those with new infections, protocol violations, and loss to follow-up were excluded from the PCR-corrected per protocol analysis and censored on the day of new infection/protocol violation/loss to follow-up in the Kaplan–Meier analysis. The efficacy and the Kaplan–Meier estimates of the cumulative risk of failure were calculated according to the per-protocol population, which included all patients fulfilling the protocol eligibility criteria, having completed the 3-day course of study medication, accomplishing the day 28 assessment and having an evaluable PCR in case of recurrent parasitemia. Statistical analyses were done at 5% of significance level using R (R Core Team 2019; R: A language and environment for statistical computing (R Foundation for Statistical Computing, Vienna, Austria)). Paired t-test was used to analyse the variation in participants’ haemoglobin over the visits (day 0, day 3, and day 28).

### Ethical considerations

The study protocol was approved on December 13, 2017, (Ref. 516/CNBS/17) by the National Bioethics Committee for Health of Mozambique (CNBS—IRB00002657). The trial was conducted according to the Good Clinical Practice guidelines. Written consent was obtained from the parents or guardians of the children who were recruited for the study. The clinical-trial identifier is NCT04370977. Staff from the CDC provided technical assistance; the protocol was approved as non-engaged research by the Centers for Global Health at the CDC (protocol # 2017-361).

## Results

### Study profile and baseline characteristics of enrolled patients

**Fig. 2:**
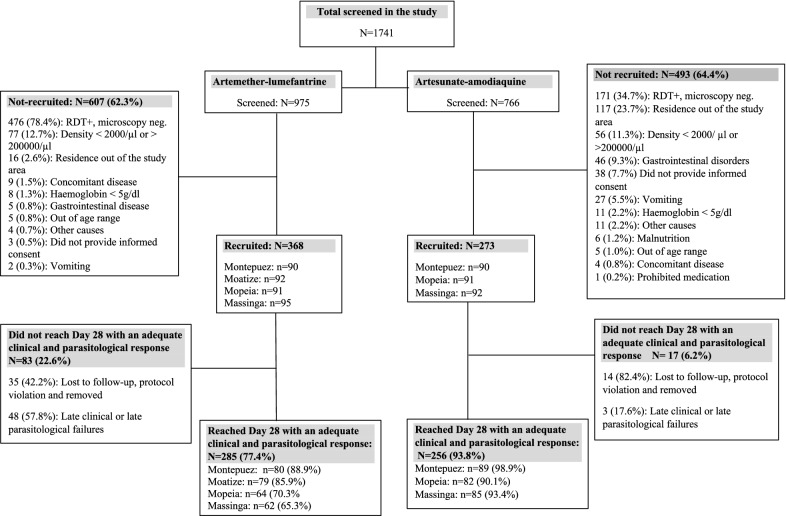
Trial profile

A total of 1741 children were screened. Of the 975 children screened in the AL arm, 368 (37.7%) were recruited and 285 (77.4%) of those recruited completed the study. For the AS–AQ arm, 766 children were screened, of whom 273 (35.6%) were recruited and 256 (93.7%) of those recruited completed the study (Fig. 2). The main reasons for exclusion were the absence of malaria parasites at the time of recruitment, parasitaemia outside the inclusion range, and living outside the study area. Table 1 summarizes the baseline characteristics of the recruited participants in both treatment arms.

**Table 1:** Baseline characteristics of study participants according to treatment arm (artemether–lumefantrine vs. artesunate–amodiaquine) at four sites in Mozambique, 2018

Artemether–lumefantrine	Study site			
	Massinga (N = 95)	Moatize (N = 92)	Montepuez (N = 90)	Mopeia (N = 91)
Female gender n (%)	42 (44.2)	45 (48.9)	46 (51.1)	38 (41.8)
Age in months (mean ± SD)	27.8 ± 14.8	34.5 ± 15.5	24 ± 15.3	30.1 ± 12.7
Weight in kg (mean ± SD)	10.7 ± 3.9	12.7 ± 3.1	9.4 ± 3.7	11.1 ± 1.9
Fever n (%)	95 (100)	92 (100)	89 (98.9)	90 (98.9)
Temperature in °C (mean ± SD)	38.4 ± 0.9	38.8 ± 1	38.3 ± 0.8	38 ± 0.6
Parasite density geometric mean (range)	24,414.2 (779–184,355)	43,265.3 (646–192,875)	46,603.7 (3060–183,724)	44,131.7 (2201–184,955)
Hb in g/dl (mean ± SD)	8.7 ± 1.8	10.2 ± 1.5	9.1 ± 1.7	9.4 ± 1.6

Artesunate–amodiaquine	Massinga (N = 92)	Moatize (N = 0)	Montepuez (N = 90)	Mopeia (N = 91)
Female gender n (%)	42 (45.7)	NA	34 (37.8)	45 (49.5)
Age in months (mean ± SD)	30.8 ± 14.4	NA	24.8 ± 14.8	30.1 ± 13.8
Weight in kg (mean ± SD)	10 ± 5.1	NA	10.5 ± 2.5	11.8 ± 2.3
Fever n (%)	92 (100)	NA	89 (98.9)	91 (100)
Temperature in °C (mean ± SD)	38.4 ± 0.9	NA	38.4 ± 0.9	37.8 ± 0.4
Parasite density geometric mean (range)	32,081.4 (1845–204,526)	NA	34,703.6 (2757–140,929)	34,293.9 (2154–173,070)
Hb in g/dl (mean ± SD)	8.9 ± 2	NA	9.5 ± 1.7	9.1 ± 1.4

AL: artemether–lumefantrine; AS–AQ: artesunate–amodiaquine; Fever: axillary temperature ≥ 37.5 °C or history of fever during the 24 h before recruitment; Hb: haemoglobin; SD: standard deviation; NA: not applicable

### Efficacy

**Fig. 3:**
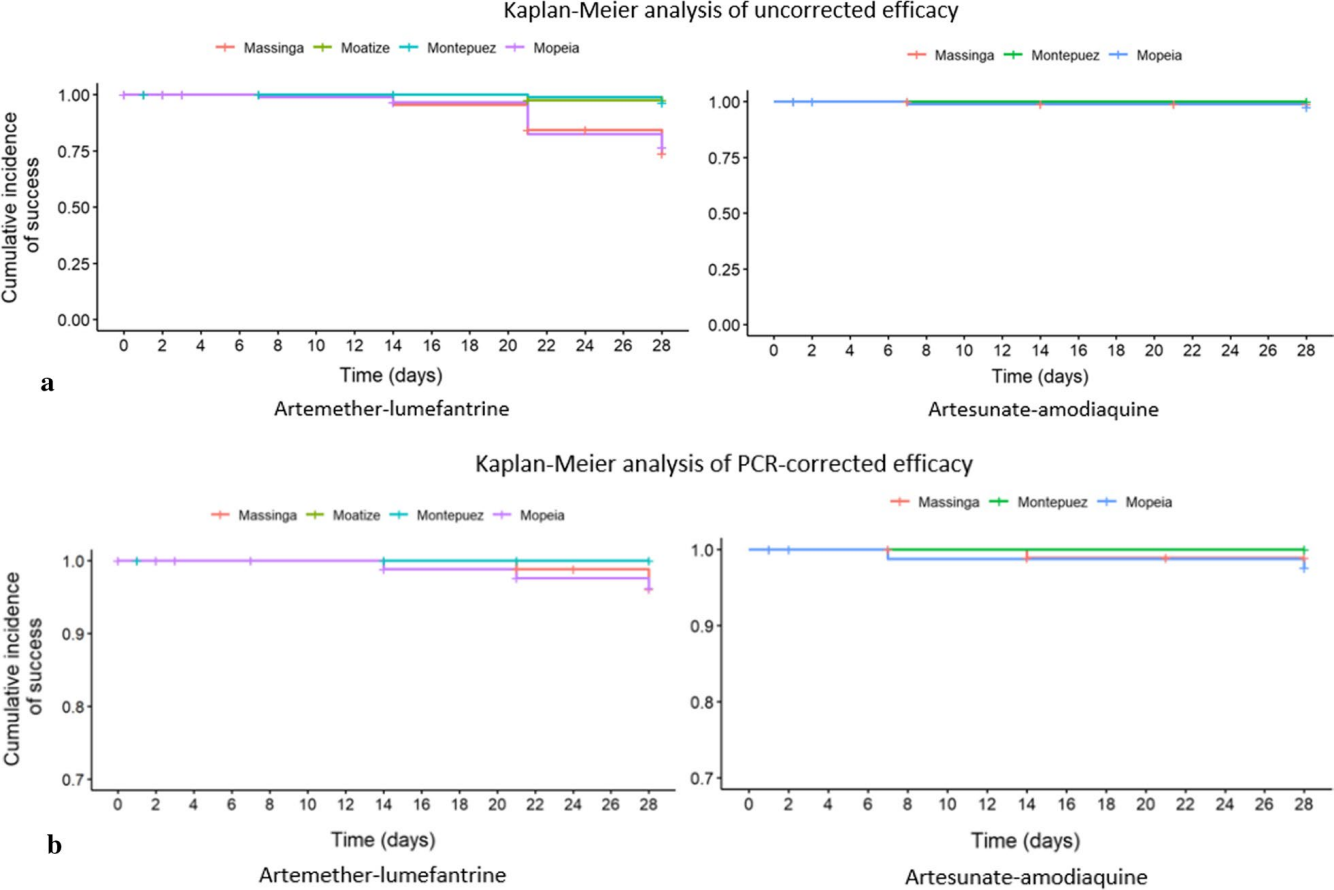
**a** Kaplan–Meier analysis of uncorrected efficacy according to the study arm. **b** Kaplan–Meier analysis of corrected efficacy according to the study arm

Table 2, Fig. 3a, b summarize treatment outcomes by study site and treatment arms. For all the sites combined, the day 28 uncorrected (crude) efficacy was 85.6% (285/333; 95% CI 81.3–89.2) for AL and 98.8% (256/259; 95% CI 96.7–99.8) for AS–AQ. Of the 48 participants with recurrent parasitaemia detected during the study, only six cases were classified as recrudescent infections (three in Mopeia and three in Massinga), making the day 28 PCR-corrected efficacy 97.9% (285/291; 95% CI 95.6–99.2) for AL (classification by genotyping can be found in Additional file 1: Table S1). In the AS–AQ arm, of the three cases with recurrent parasitaemia, one was classified as a new infection and another as recrudescent infection in Mopeia, and the last one in Massinga was indeterminate by genotyping, so the combined day 28 PCR-corrected for AS–AQ was calculated as 99.6% (256/257; 95% CI 97.9–100; Additional file 1: Table S1).

**Table 2:** Treatment outcome on day 28, according to treatment arm at four sites in Mozambique, 2018

Artemether–lumefantrine	Study site				
	Massinga (N = 95)	Moatize (N = 92)	Montepuez (N = 90)	Mopeia (N = 91)	Total (N = 368)
ACPR (uncorrected) n	62	79	80	64	285
ETF	0	0	0	0	0
LCF	8	0	0	15	23
LPF	15	2	3	5	25
New infections (with PCR)	20	2	3	17	42
Recrudescences (with PCR)	3	0	0	3	6
Undetermined (with PCR)	0	0	0	0	0
No treatment outcome (lost to follow-up or withdrawn)	10	11	7	7	35
PP day 28 efficacy (PCR-uncorrected) n/N (%) [95%CI]	62/85 (72.9) [62.2–82.0]	79/81 (97.5) [91.4–99.7]	80/83 (96.4) [89.8–99.2]	64/84 (76.2) [65.6–84.8]	285/333 (85.6) [81.3–89.2]
PP day 28 efficacy (PCR-corrected) n/N (%) [95%CI]	62/65 (95.4) [87.1–99]	79/79 (100) [95.4–100]	80/80 (100) [95.5–100]	64/67 (95.5) [87.5–99.1]	285/291 (97.9) [95.6–99.2]

Artesunate–amodiaquine	Massinga (N = 92)	Moatize (N = 0)	Montepuez (N = 90)	Mopeia (N = 91)	Total (N = 273)
ACPR (uncorrected) n	85	NA	89	82	256
ETF	0	NA	0	0	0
LCF	0	NA	0	2	2
LPF	1	NA	0	0	1
New infections (with PCR)	0	NA	0	1	1
Recrudescences (with PCR)	0	NA	0	1	1
Undetermined (with PCR)	1	NA	0	0	1
No treatment outcome (loss to follow-up or withdrawn)	6	NA	1	7	14
PP day 28 efficacy (PCR-uncorrected) n/N (%) [95%CI]	85/86 (98.8) [93.7–100]	NA	89/89 (100) [95.9–100]	82/84 (97.6) [91.7–99.7]	256/259 (98.8) [96.7–99.8]
PP day 28 efficacy (PCR-corrected) n/N (%) [95%CI]	85/85 (100) [95.8–100]	NA	89/89 (100) [95.9–100]	82/83 (98.8) [93.5–100]	256/257 (99.6) [97.9–100]

*ACPR* adequate clinical and parasitological response, *ETF* early treatment failure, *LCF* late clinical failure, *LPF* late parasitological failure, *PP* per protocol, *PCR* polymerase chain reaction, *NA* not applicable

### Parasitemia clearance

**Fig. 4:**
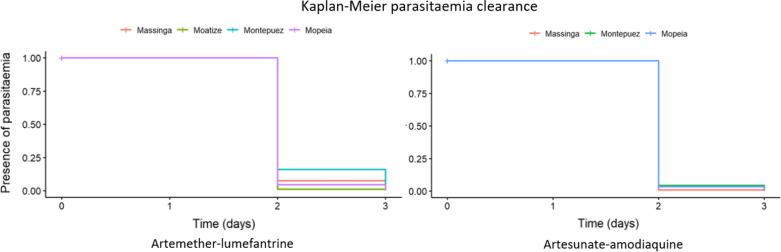
Kaplan–Meier curve showing time to negative parasitemia according to the study site, treatment arm and day of follow-up

**Fig. 5:**
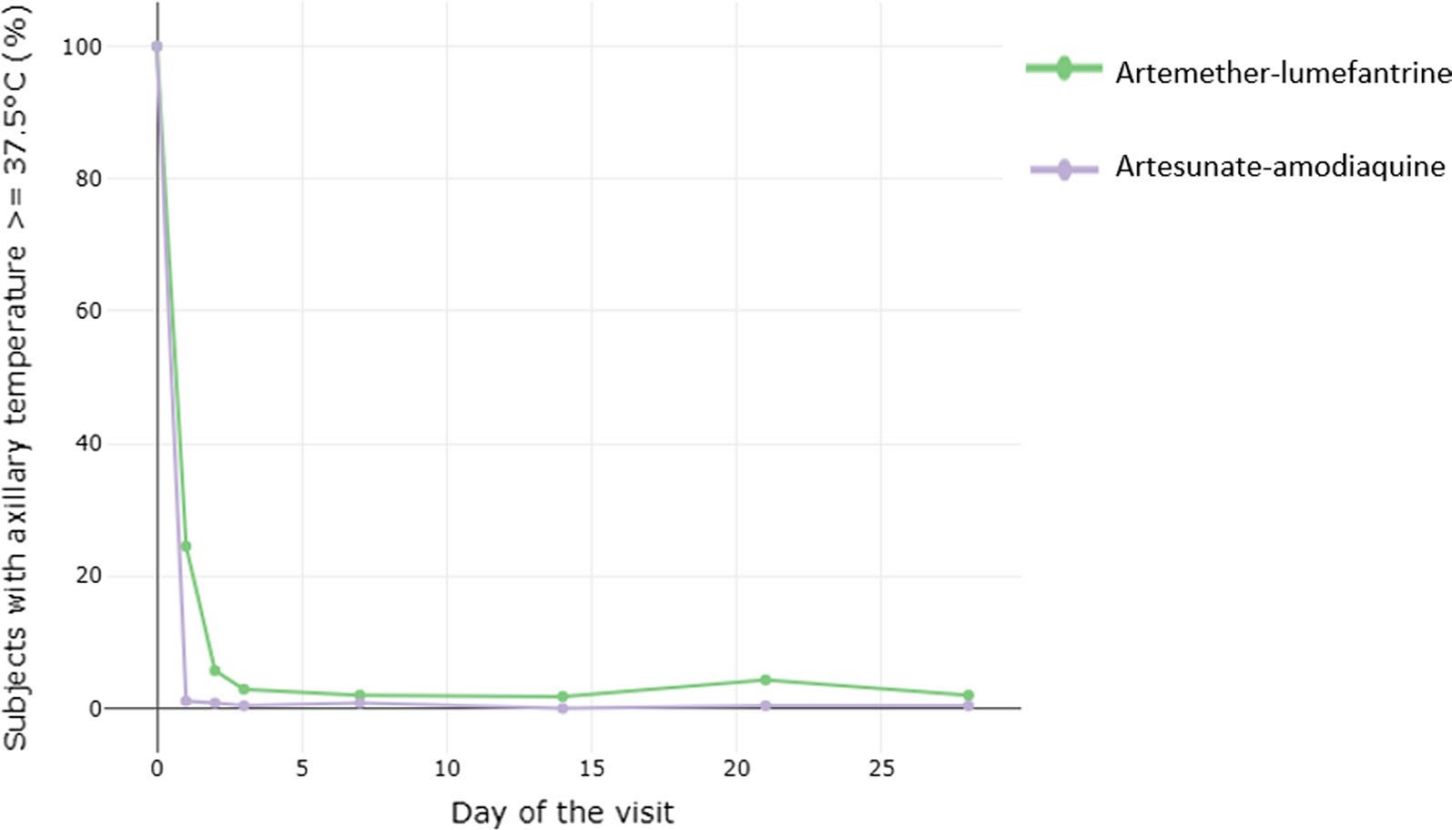
Percentage of children with fever according to the follow-up day and study arm

The majority of the patients in both arms cleared their parasitaemia by day 3, however, 7.1% (26/368) for AL and 3.0% (8/273) for AS–AQ remained parasitaemic by day 3 (Fig. 4). Most patients, 94.3% (347/368) for AL and 96.0% (262/273) for AS–AQ, were afebrile by day 3 (Fig. 5).

### Safety and tolerability

Table 3 summarizes the main adverse events observed during the first 3 days of treatment. During the study, five serious adverse events were documented in the AL arm: two patients with persistent vomiting within the first 30 min of treatment on day 0, one patient hospitalized after two doses with febrile seizures, and two patients hospitalized for severe malaria (the first, 1 week after recruitment; the second, 2 weeks after recruitment). In each case, it was necessary to stop their involvement in the study and switch to the rescue treatment, which resulted in resolution for all five cases. None of these adverse events was considered by the investigators to be related to the study drug.

**Table 3:** Adverse events reported during 3 days of treatment according to treatment arm in four sites in Mozambique, 2018

Artemether–lumefantrine	Study site				
	Massinga (N = 95)	Moatize (N = 92)	Montepuez (N = 90)	Mopeia (N = 91)	Total (N = 368)
Vomiting n (%)	1 (1.1)	1 (1.1)	1 (1.1)	0 (0)	3 (0.8)
Diarrhoea n (%)	0 (0)	0 (0)	1 (1.1)	0 (0)	1 (0.3)
Weakness n (%)	0 (0)	0 (0)	1 (1.1)	1 (1.1)	2 (0.5)
Dizziness n (%)	0 (0)	1 (1.1)	3 (3.3)	1 (1.1)	5 (1.4)
Fainting n (%)	0 (0)	0 (0)	1 (1.1)	1 (1.1)	2 (0.5)
Pruritus n (%)	0 (0)	0 (0)	1 (1.1)	1 (1.1)	2 (0.5)
Urticaria n (%)	0 (0)	1 (1.1)	3 (3.3)	3 (3.3)	7 (1.9)

Artesunate–amodiaquine	Massinga (N = 92)	Moatize (N = 0)	Montepuez (N = 90)	Mopeia (N = 91)	Total (N = 273)
Vomiting n (%)	1 (1.1)	NA	1 (1.1)	0 (0)	2 (0.7)
Diarrhoea n (%)	1 (1.1)	NA	1 (1.1)	0 (0)	2 (0.7)
Weakness n (%)	0 (0)	NA	1 (1.1)	0 (0)	1 (0.4)
Dizziness n (%)	0 (0)	NA	1 (1.1)	0 (0)	1 (0.4)
Fainting n (%)	0 (0)	NA	2 (2.2)	0 (0)	2 (0.7)
Pruritus n (%)	0 (0)	NA	1 (1.1)	0 (0)	1 (0.4)
Urticaria n (%)	1 (1.1)	NA	1 (1.1)	0 (0)	2 (0.7)

*NA* not applicable

### Haemoglobin recovery and gametocyte clearance

**Fig. 6:**
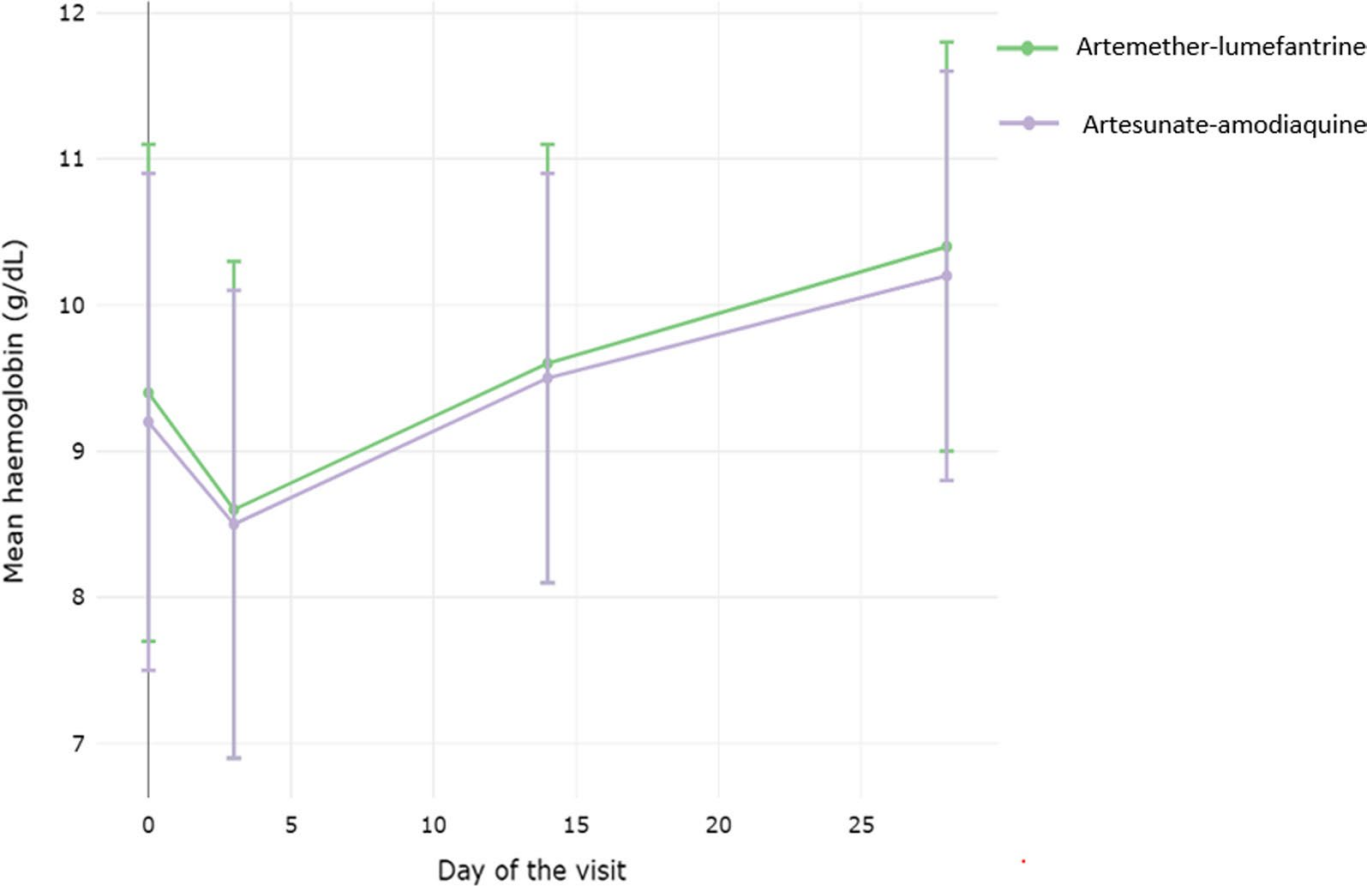
Changes in mean haemoglobin according to the study arm

Overall, the mean haemoglobin levels dropped from 9.4 g/dl on day 0 to 8.6 g/dl on day 3 [mean decrease 0.8 (SD 1.7)] in the AL arm (*P* = 0.0002) and from 9.2 to 8.5 g/dl [mean decrease 0.7 (SD 1.7)] in the AS–AQ arm (*P* = 0.0003) but recovered after treatment, from day 3 to day 28 [mean increase 1.8 (SD 1.6)] in the AL arm (*P* = 0.002) and [mean increase 1.7 (SD 1.5)] in the AS–AQ arm (*P* = 0.002) using paired t-test (Fig. 6). Gametocytes were only observed in 13 patients on day 0, nine of these were in the AS–AQ arm (one in Massinga and eight in Mopeia) and four were in the AL arm (three in Massinga and one in Moatize). All 13 patients cleared their gametocytes during the follow-up period, although another patient in the AL arm (new infection) had detectable gametocytes on 28 day in Moatize (Additional file 2: Figure S1).

## Discussion

Because of the high burden of malaria in Mozambique, it remains critical to have effective antimalarial treatments and for periodic in vivo studies to evaluate their efficacy. The overall PCR-corrected efficacy of AL and AS–AQ, anti-malarials currently used in Mozambique, remains above 95% in the four sites investigated in this study. Although AS–AQ is the co-first line therapy, this regimen is infrequently used in the country, with AL being the most prescribed treatment. Based on comparison to results from previous studies conducted in Mozambique, the efficacy of AL has remained relatively unchanged over time, with a 96.0% PCR-corrected efficacy in 2011–2012 [12], 98.4% in 2015 [13], and. 97.9% in the present study. In Montepeuz, the one site that has remained constant over these three studies, the PCR-corrected efficacy of AL was 94.2% in 2011–2012, 98.7% in 2015, and 100% in the present study. Collectively, these results support the continued use of AL for malaria in Mozambique.

In this study, a Bayesian approach was used to distinguish between new and recrudescent infections. This is especially important in high transmission areas such as Mozambique, where a high multiplicity of infection is likely. A high multiplicity of infection can increase the likelihood of shared alleles between day 0 and day of recurrent infections and often low-frequency alleles may be missed, leading to misclassification bias during PCR-correction. Using a Bayesian approach that accounts for the frequency of alleles in the entire sample may have reduced this likelihood.

A higher percentage of patients in the AL arm had recurring parasitaemia (48/333, 14.4%) compared to the AS–AQ arm (3/259, 1.2%). These findings are similar to findings found in previous studies conducted in Mozambique and other parts of Africa [12, 23, 24]. Several factors can explain recurrent infections during a therapeutic efficacy study, including transmission intensity of the study site (with more recurrent infections occurring in higher than lower transmission settings), the effectiveness and elimination half-life of the partner drugs, and host factors, such as drug absorption and metabolism [17]. In general, effective partner drugs provide a prophylactic effect due to their longer half-life compared to artemisinin derivatives. The higher number of recurrent infections observed in the AL arm may be directly related to the much longer post-treatment prophylactic effect exerted by the longer half-life of amodiaquine (~ 9–18 days) [25, 26] than for lumefantrine (~ 4–5 days) [27]. One important caveat is that the drug arms were sequential at each site (except Moatize, where only AL was evaluated). In other words, both ACTs were not tested at the same time of the season. Throoughout Mozambique, the peak malaria season usually occurs between November and April. Therefore, different transmission intensities may have been present for the AL arm (which was conducted between February and June in all four sites) and the AS–AQ arm (which was conducted between June and September in the three sites where AS–AQ was evaluated), precluding a definitive comparison of reinfection rates between the two ACTs. Another consideration for future studies would be to collect drug levels, which could further explain higher reinfection rates in a particular arm.

While many factors can affect the day 3 positivity of treated patients, measuring day 3 positivity during a therapeutic efficacy study and determining its changes over time in a country is an important parameter to determine the efficacy of the artemisinin component. A positivity of > 10% is a criterion of artemisinin partial resistance [28]. In this study, the positivity rate on day 3 was 7% for AL and 3% for AS–AQ. This was higher than previously reported (< 2%) [12, 13] but below the 10% threshold for suspected artemisinin partial resistance [28]. Therefore, while no change in the treatment policy is needed, heightened vigilance is necessary to ascertain the future efficacy of AL in Mozambique. These findings showed that both AL and AS–AQ were effective in ultimately clearing gametocytes, similar to previous studies [13, 29]. However, there was a higher percentatge of subjects with detectable gametocytes at days 3 and 7 in those treated with AS–AQ than AL, a finding consistent with a multicenter analysis incorporating studies from 16 sub-Saharan African countries [30]. Both drugs showed a rapid recovery of haemoglobin after treatment, consistent with other studies previously conducted in Mozambique [12, 13]. In addition, both drugs were well-tolerated.

## Conclusion

This study indicates that both AL and AS–AQ are still efficacious for the treatment of uncomplicated malaria and remain very well-tolerated in Mozambique. Monitoring their efficacy, including anti-malarial resistance molecular markers, every 2 years, as recommended by the WHO, should continue.

## Supplementary Information

**Additional file 1: Table S1.** Observed fragment lengths of neutral microsatellite loci from paired Day 0 (D0) and Day of Failure samples (D7, D14, D21, etc.) from a therapeutic efficacy study in Mozambique, 2018.

**Additional file 2: Figure S1.** Percentage of children with gametocytes according to treatment arm and day of follow-up.

## Data Availability

The datasets used during the current study are available from the corresponding author on reasonable request.

## References

[1] WHO. World malaria report 2020: 20 years of global progress and challenges. Geneva: World Health Organization; 2020. Licence: CC BY-NC-SA 3.0 IGO.

[2] Instituto Nacional de Saúde (INS) and ICF. Inquérito Nacional sobre Indicadores de Malária em Moçambique 2018. Maputo, Moçambique. Rockville, USA. 2019.

[3] Noedl H, Se Y, Schaecher K, Smith BL, Socheat D, Fukuda MM (2008). Evidence of artemisinin-resistant malaria in Western Cambodia. N Engl J Med.

[4] Dondorp AM, Nosten F, Yi P, Das D, Phyo AP, Tarning J (2009). Artemisinin resistance in *Plasmodium falciparum* malaria. N Engl J Med.

[5] Noedl H, Socheat D, Satimai W (2009). Artemisinin-resistant malaria in Asia. N Engl J Med.

[6] Leang R, Taylor WRJ, Bouth DM, Song L, Tarning J, Char MC (2015). Evidence of *Plasmodium falciparum* malaria multidrug resistance to artemisinin and piperaquine in Western Cambodia: dihydroartemisinin–piperaquine open-label multicenter clinical assessment. Antimicrob Agents Chemother.

[7] Ariey F, Witkowski B, Amaratunga C, Beghain J, Langlois A-C, Khim N (2014). A molecular marker of artemisinin-resistant *Plasmodium falciparum* malaria. Nature.

[8] Uwimana A, Umulisa N, Venkatesan M, Svigel SS, Zhou Z, Munyaneza T (2021). Association of *Plasmodium falciparum* kelch13 R561H genotypes with delayed parasite clearance in Rwanda: an open-label, single-arm, multicentre, therapeutic efficacy study. Lancet Infect Dis.

[9] Abacassamo F, Enosse S, Aponte JJ, Gómez-Olivé FX, Quintó L, Mabunda S (2004). Efficacy of chloroquine, amodiaquine, sulphadoxine–pyrimethamine and combination therapy with artesunate in Mozambican children with non-complicated malaria. Trop Med Int Health.

[10] Bassat Q, Mulenga M, Tinto H, Piola P, Borrmann S, Nabasumba C (2009). Lumefantrine for treating uncomplicated malaria in African children : a randomised, non-inferiority trial. PLoS ONE.

[11] Abdulla S, Sagara I, Borrmann S, D’Alessandro U, Gonzalez R, Hamel M (2008). Efficacy and safety of artemether–lumefantrine dispersible tablets compared with crushed commercial tablets in African infants and children with uncomplicated malaria: a randomised, single-blind, multicentre trial. Lancet.

[12] Nhama A, Bassat Q, Enosse S, Nhacolo A, Mutemba R, Carvalho E (2014). In vivo efficacy of artemether-lumefantrine and artesunate-amodiaquine for the treatment of uncomplicated falciparum malaria in children: a multisite, open-label, two-cohort, clinical trial in Mozambique. Malar J.

[13] Salvador C, Rafael B, Matsinhe F, Candrinho B, Muthemba R, De Carvalho E (2017). Efficacy and safety of artemether–lumefantrine for the treatment of uncomplicated falciparum malaria at sentinel sites in Mozambique, 2015. Acta Trop.

[14] Bassat Q, Gonzalez R, Machevo S, Nahum A, Lyimo J, Maiga H (2011). Similar efficacy and safety of artemether-lumefantrine (Coartem(R)) in African infants and children with uncomplicated falciparum malaria across different body weight ranges. Malar J.

[15] WHO. Methods for surveillance of antimalarial drug efficacy: genotyping to identify parasite populations. Geneva: World Health Organization; 2009. www.who.int.

[16] WHO (2000). Severe falciparum malaria. Trans R Soc Trop Med Hyg.

[17] WHO. Guidelines for the treatment of malaria. 3rd edition. Geneva: World Health Organization; 2015. http://www.who.int/malaria/publications. 26020088

[18] MISAU/DNSP/PNCM. Normas de Tramento da malária em Moçambique. Maputo, Moçambique. 2017. www.telesaude.co.mz.

[19] Halsey ES, Venkatesan M, Plucinski MM, Talundzic E, Lucchi NW, Zhou Z (2017). Capacity development through the US President’s Malaria Initiative-supported antimalarial resistance monitoring in Africa Network. Merg Infect Dis.

[20] Lucchi NW, Narayanan J, Karell MA, Xayavong M, Kariuki S, DaSilva AJ (2013). Molecular diagnosis of malaria by photo-induced electron transfer fluorogenic primers: PET-PCR. PLoS ONE.

[21] Anderson TJ, Haubold B, Williams JT, Estrada-Franco JG, Richardson L, Mollinedo R (2000). Microsatellite markers reveal a spectrum of population structures in the malaria parasite *Plasmodium falciparum*. Mol Biol Evol.

[22] Plucinski MM, Morton L, Bushman M, Dimbu PR, Udhayakumar V (2015). Robust algorithm for systematic classification of malaria late treatment failures as recrudescence or reinfection using microsatellite genotyping. Antimicrob Agents Chemother.

[23] Paczkowski M, Mwandama D, Marthey D, Luka M, Makuta G, Sande J (2016). In vivo efficacy of artemether-lumefantrine and artesunate-amodiaquine for uncomplicated *Plasmodium falciparum* malaria in Malawi, 2014. Malar J.

[24] Plucinski MM, Dimbu PR, Macaia AP, Ferreira CM, Samutondo C, Quivinja J (2017). Efficacy of artemether-lumefantrine, dihydroartemisinin-piperaquine for treatment of uncomplicated *Plasmodium falciparum* malaria in Angola, 2015. Malar J.

[25] Stepniewska K, Taylor W, Sirima SB, Ouedraogo EB, Ouedraogo A, Gansané A (2009). Population pharmacokinetics of artesunate and amodiaquine in African children. Malar J.

[26] Hombhanje FW, Hwaihwanje I, Tsukahara T, Saruwatari J, Nakagawa M, Osawa H (2005). The disposition of oral amodiaquine in Papua New Guinean children with falciparum malaria. Br J Clin Pharmacol.

[27] Staehli Hodel EM, Guidi M, Zanolari B, Mercier T, Duong S, Kabanywanyi AM (2013). Population pharmacokinetics of mefloquine, piperaquine and artemether-lumefantrine in Cambodian and Tanzanian malaria patients. Malar J.

[28] WHO. Report on antimalarial drug efficacy, resistance and response: 10 years of surveillance (2010–2019). Geneva: World Health Organization; 2020. Licence: CC BY-NC-SA 3.0 IGO.

[29] Abuaku B, Duah N, Quaye L, Quashie N, Malm K, Plange CB (2016). Therapeutic efficacy of artesunate-amodiaquine and artemether-lumefantrine combinations in the treatment of uncomplicated malaria in two ecological zones in Ghana. Malar J.

[30] Zwang J, Olliaro P, Barennes H, Bonnet M, Brasseur P, Bukirwa H (2009). Efficacy of artesunate-amodiaquine for treating uncomplicated falciparum malaria in sub-Saharan Africa: a multi-centre analysis. Malar J.

