# Supplementary material for: In vivo efficacy and safety of artemether–lumefantrine and amodiaquine–artesunate for uncomplicated Plasmodium falciparum malaria in Mozambique, 2018
Source: Malar J. 2021 Oct 2;20:390. doi: 10.1186/s12936-021-03922-9 (PMC8487544; doi:10.1186/s12936-021-03922-9)
Supplement: Supplementary file 2 — Additional file 2: Figure S1. Percentage of children with gametocytes according to treatment arm and day of follow-up. [file 12936_2021_3922_MOESM2_ESM.docx]

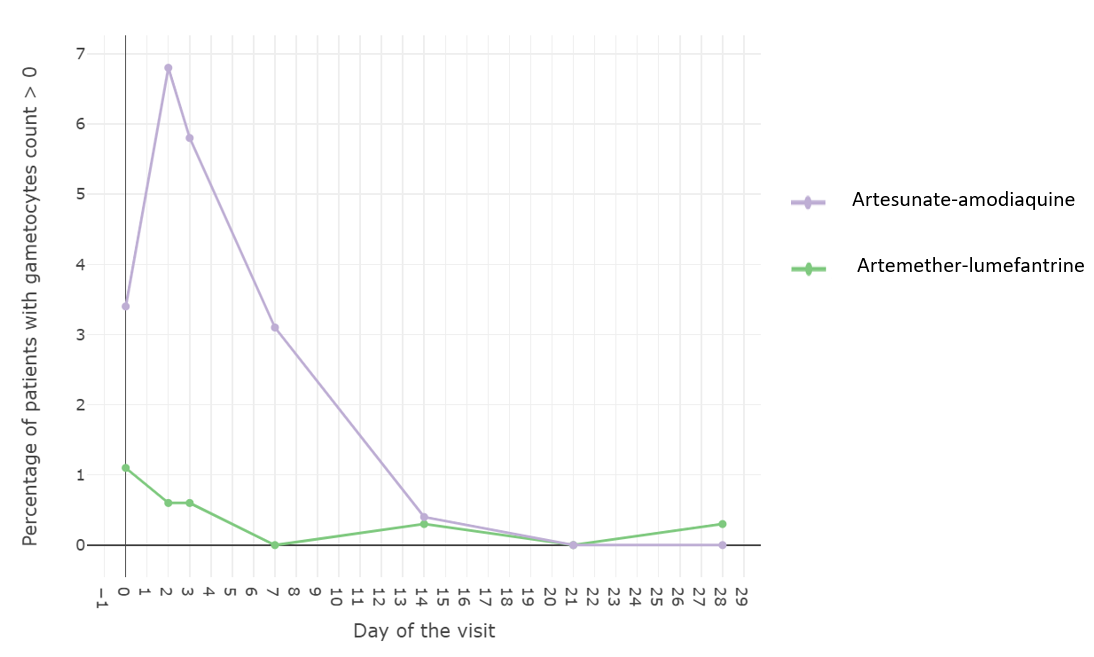


**Additional file 2: Figure S1**: Percentage of children with gametocytes according to treatment arm and day of follow-up.
